# Long-term survival in patients treated with ruxolitinib for myelofibrosis: COMFORT-I and -II pooled analyses

**DOI:** 10.1186/s13045-017-0527-7

**Published:** 2017-09-29

**Authors:** Srdan Verstovsek, Jason Gotlib, Ruben A. Mesa, Alessandro M. Vannucchi, Jean-Jacques Kiladjian, Francisco Cervantes, Claire N. Harrison, Ronald Paquette, William Sun, Ahmad Naim, Peter Langmuir, Tuochuan Dong, Prashanth Gopalakrishna, Vikas Gupta

**Affiliations:** 1 The University of Texas MD Anderson Cancer Center, Division of Cancer Medicine, 1515 Holcombe Blvd, Unit 418, Houston, TX 77030 USA; 2 Stanford Cancer Institute, Stanford, CA USA; 3 UT Health San Antonio Cancer Center – An NCI Designated Cancer Center, San Antonio, TX USA; 4 Center for Research and Innovation of Myeloproliferative Neoplasms, AOU Careggi, and Laboratorio Congiunto, University of Florence, Florence, Italy; 5 Centre d’Investigations Cliniques (INSERM CIC 1427), Hôpital Saint-Louis and Université Paris Diderot, Paris, France; 6 Hospital Clínic, Institut d’Investigacions Biomèdiques August Pi i Sunyer, Barcelona, Spain; 7 Guy’s and St. Thomas’ NHS Foundation Trust, London, UK; 8 Cedars-Sinai Medical Center, Los Angeles, CA USA; 9 Incyte Corporation, Wilmington, DE USA; 10 Novartis Pharmaceutical Corporation, East Hanover, NJ USA; 11 Novartis Pharma AG, Basel, Switzerland; 12 Princess Margaret Cancer Centre, University of Toronto, Toronto, ON Canada

**Keywords:** Ruxolitinib, Myelofibrosis, Overall survival, Anemia, Transfusion

## Abstract

**Background:**

Myelofibrosis (MF) is associated with a variety of burdensome symptoms and reduced survival compared with age-/sex-matched controls. This analysis evaluated the long-term survival benefit with ruxolitinib, a Janus kinase (JAK)1/JAK2 inhibitor, in patients with intermediate-2 (int-2) or high-risk MF.

**Methods:**

This was an exploratory analysis of 5-year data pooled from the phase 3 COMFORT-I and -II trials. In both trials, patients could cross over to ruxolitinib from the control group (COMFORT-I, placebo; COMFORT-II, best available therapy). All continuing patients in the control groups crossed over to ruxolitinib by the 3-year follow-up. Overall survival (OS; a secondary endpoint in both trials) was evaluated using pooled intent-to-treat data from patients randomized to ruxolitinib or the control groups. OS was also evaluated in subgroups stratified by baseline anemia and transfusion status at week 24.

**Results:**

A total of 528 patients were included in this analysis; 301 were originally randomized to ruxolitinib (COMFORT-I, *n* = 155; COMFORT-II, *n* = 146) and 227 to control (*n* = 154 and *n* = 73, respectively). The risk of death was reduced by 30% among patients randomized to ruxolitinib compared with patients in the control group (median OS, 5.3 vs 3.8 years, respectively; hazard ratio [HR], 0.70 [95% CI, 0.54–0.91]; *P* = 0.0065). After correcting for crossover using a rank-preserving structural failure time (RPSFT) method, the OS advantage was more pronounced for patients who were originally randomized to ruxolitinib compared with patients who crossed over from control to ruxolitinib (median OS, 5.3 vs 2.3 years; HR [ruxolitinib vs RPSFT], 0.35 [95% CI, 0.23–0.59]). An analysis of OS censoring patients at the time of crossover also demonstrated that ruxolitinib prolonged OS compared with control (median OS, 5.3 vs 2.4 years; HR [ruxolitinib vs censored at crossover], 0.53 [95% CI, 0.36–0.78]; *P* = 0.0013). The survival benefit with ruxolitinib was observed irrespective of baseline anemia status or transfusion requirements at week 24.

**Conclusions:**

These findings support ruxolitinib treatment for patients with int-2 or high-risk MF, regardless of anemia or transfusion status. Further analyses will be important for exploring ruxolitinib earlier in the disease course to assess the effect on the natural history of MF.

**Trial registration:**

ClinicalTrials.gov identifiers, NCT00952289 and NCT00934544.

## Background

Myelofibrosis (MF) is associated with progressive bone marrow fibrosis, splenomegaly [1], burdensome symptoms [2], and reduced survival compared with age- and sex-matched controls [3]. Anemia [4] and transfusion dependence [5] are associated with shortened overall survival (OS) in patients with MF. However, the effects of transfusion status on OS have not been evaluated in patients receiving ruxolitinib.

Ruxolitinib is a Janus kinase (JAK)1/JAK2 inhibitor approved by the European Medicines Agency for the treatment of disease-related splenomegaly or symptoms in adult patients with primary MF (PMF), post–polycythemia vera MF (PPV-MF), and post–essential thrombocythemia MF (PET-MF) [6], and by the US Food and Drug Administration for intermediate or high-risk MF, including PMF, PPV-MF, and PET-MF [7]. Primary results from two long-term, pivotal phase 3 clinical trials (COMFORT-I and COMFORT-II) demonstrated that ruxolitinib reduced spleen volume, improved MF-related symptoms and quality-of-life measures, and was associated with prolonged OS in patients with intermediate-2 (int-2) or high-risk MF compared with controls [8–10]. Exploratory analyses of pooled 3-year data from the COMFORT trials showed that OS favored ruxolitinib regardless of baseline anemia status or development of new or worsening anemia post baseline [11].

Here, we report the findings from exploratory analyses of long-term OS benefit with ruxolitinib using pooled 5-year data from the COMFORT trials. In addition, OS was also evaluated in subgroups stratified by baseline anemia and week 24 transfusion status.

## Methods

The double-blind COMFORT-I and open-label COMFORT-II trials (ClinicalTrials.gov identifiers, NCT00952289, NCT00934544) were randomized phase 3 studies described previously [8, 10]. Briefly, all patients were ≥ 18 years of age with int-2 or high-risk PMF, PPV-MF, or PET-MF. The ruxolitinib starting dose was 15 or 20 mg twice daily based on baseline platelet counts (100–200 or > 200 × 10^9^/L, respectively). Dose modifications were permitted for safety and efficacy. Patients could cross over to ruxolitinib from the control group for progressive splenomegaly (COMFORT-I, ≥ 25% increase in spleen volume from baseline; COMFORT-II, study nadir) or for select protocol-defined progression events; crossover was mandatory following treatment unblinding in COMFORT-I. The control group in COMFORT-I received placebo. The control group in COMFORT-II received best available therapy; the three most common were hydroxyurea (47%), no medication (33%), and prednisone/prednisolone (12%). All continuing patients in the control groups crossed over to ruxolitinib by the 3-year follow-up [9, 12].

This report includes exploratory analyses of OS (a secondary endpoint in both studies) using pooled intent-to-treat (ITT) data from patients randomized to ruxolitinib and the control groups. OS was also evaluated in subgroups stratified by baseline anemia and transfusion status at week 24, defined as follows:

*Baseline anemia:* receiving any units of red blood cells (RBCs) within 12 weeks before baseline measurement or having baseline hemoglobin < 10 g/dL.

*Baseline nonanemic:* not meeting criteria for anemia.

*Transfusion independence at week 24:* absence of RBC transfusions and hemoglobin levels ≥ 8 g/dL during weeks 13 to 24.

*Not transfusion independent at week 24:* requiring RBC transfusions or hemoglobin levels < 8 g/dL during weeks 13 to 24.

*Transfusion dependence at week 24:* requiring ≥ 4 units of RBCs or hemoglobin levels < 8 g/dL during weeks 17 to 24.

*Not transfusion dependent at week 24:* requiring < 4 units of RBCs and hemoglobin levels ≥ 8 g/dL during weeks 17 to 24.

Transfusion independence/dependence subgroup status was defined separately from baseline transfusion status (e.g., patients who were transfusion independent at week 24 did not necessarily require RBC transfusions before baseline). Two analyses were performed based on the definitions of independence and dependence, comparing patients who were (1) transfusion independent at week 24 versus not independent and (2) transfusion dependent at week 24 versus not dependent.

Overall survival was evaluated using a stratified log-rank test and Cox proportional hazards model that estimated the treatment effect stratified by clinical trial and International Prognostic Scoring System (IPSS) risk [4]. The crossover-corrected treatment effect was estimated using a rank-preserving structural failure time (RPSFT) method and through censorship of survival time at the time of crossover. The effect of transfusion status on OS was evaluated using the Landmark approach, which included patients completing ≥ 24 weeks of study treatment, and the stratified log-rank test, which included study, IPSS risk, and baseline anemia status as stratification variables.

## Results

### Disposition and baseline anemia

This pooled analysis included 528 patients; 301 were originally randomized to ruxolitinib (COMFORT-I, *n* = 155; COMFORT-II, *n* = 146) and 227 were randomized to control (*n* = 154 and *n* = 73, respectively). Most patients in the control group crossed over to ruxolitinib during the study (69.6%). At data cutoff, 27.2% of patients in the ruxolitinib group versus 0 in the control group remained on treatment. Similar proportions of patients in each pooled treatment group had anemia at baseline (ruxolitinib, 45.8%; control, 49.8%); 39.3 and 67.5% of patients in the ruxolitinib and control groups, respectively, had ≥ 1 transfusion in the 28 days before baseline.

### Overall survival: ITT analysis and subgroups by IPSS risk status

**Fig. 1:**
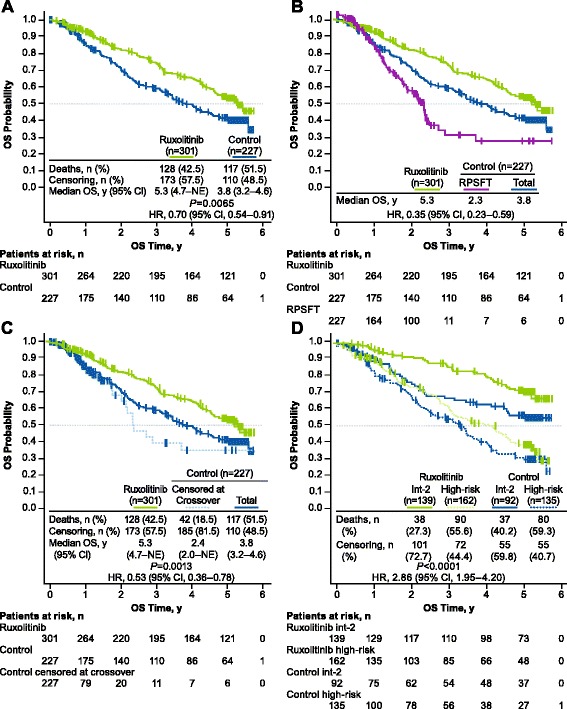
Overall survival: 5-year pooled data. OS analysis of 5-year pooled data from the COMFORT-I and -II trials. Data are presented **a** for the ITT population, **b** corrected for crossover with the RPSFT model, **c** censored at crossover, and **d** stratified by IPSS risk status. Originally presented at the American Society of Hematology 58th Annual Meeting [13]. HR, hazard ratio; int-2, intermediate-2; IPSS, International Prognostic Scoring System; ITT, intent-to-treat; NE, not evaluable; OS, overall survival; RPSFT, rank-preserving structural failure time

At the 5-year ITT analysis, 128 patients (42.5%) in the ruxolitinib group had died compared with 117 (51.5%) in the control group. The risk of death was reduced by 30% among patients randomized to ruxolitinib compared with patients in the control group (median OS, 5.3 vs 3.8 years, respectively; hazard ratio [HR; ruxolitinib vs control], 0.70 [95% CI, 0.54–0.91]; *P* = 0.0065; Fig. 1a [13]). After correcting for crossover using RPSFT, the OS advantage was more pronounced in patients who were originally randomized to ruxolitinib compared with patients who crossed over from control to ruxolitinib (median OS, 5.3 vs 2.3 years; HR [ruxolitinib vs RPSFT], 0.35 [95% CI, 0.23–0.59]; Fig. 1b). An analysis of OS censoring patients at the time of crossover also demonstrated that ruxolitinib prolonged survival compared with control (median OS, 5.3 vs 2.4 years; HR [ruxolitinib vs censored at crossover], 0.53 [95% CI, 0.36–0.78]; *P* = 0.0013; Fig. 1c).

Among patients randomized to ruxolitinib, those with int-2 MF had longer median OS than those with high-risk MF (median OS, int-2 not reached, estimated, 8.5 years; high-risk, 4.2 years; HR [high vs int-2], 2.86 [95% CI, 1.95–4.20]; *P* < 0.0001; Fig. 1d).

### Overall survival: subgroups by baseline anemia status and week 24 transfusion status

**Fig. 2:**
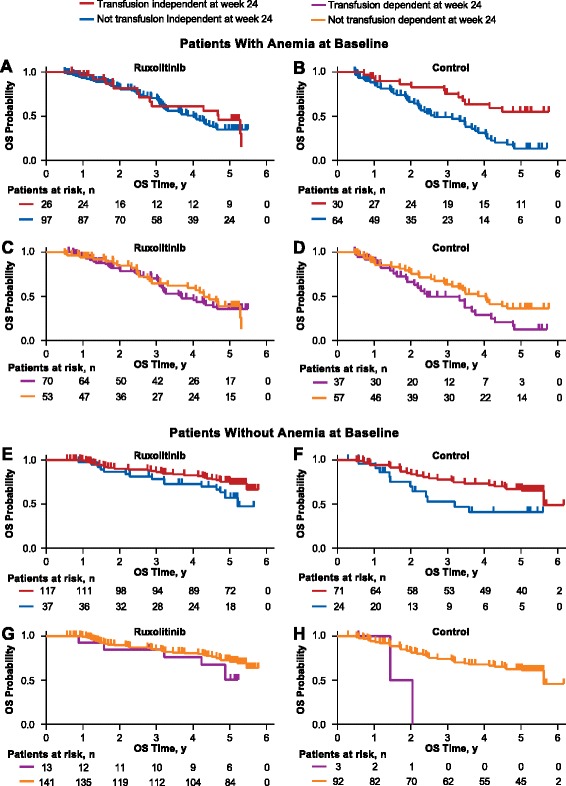
Overall survival: 5-year pooled data stratified by baseline anemia status and week 24 transfusion status. OS analysis of 5-year pooled data from the COMFORT-I and -II trials stratified by baseline anemia status and week 24 transfusion status. Patients in the ruxolitinib and control groups were stratified by anemia status at baseline and (**a**, **b**, **e**, **f**) transfusion independence status at week 24 or (**c**, **d**, **g**, **h**) transfusion dependence status at week 24. OS probability in the ruxolitinib group was not significantly affected by transfusion status at week 24 (transfusion independent vs not independent, *P* = 0.1322*; transfusion dependent vs not dependent, *P* = 0.4547*), but was significantly affected in the control group (transfusion independent vs not independent, *P* = 0.0004*; transfusion dependent vs not dependent, *P* = 0.0323*). Baseline anemia was defined as receiving any units of RBCs within 12 weeks before baseline measurement or having baseline hemoglobin < 10 g/dL; nonanemic was defined as not meeting criteria for anemia. Transfusion independence at week 24 was defined as the absence of RBC transfusions and hemoglobin levels ≥ 8 g/dL during weeks 13 to 24; not transfusion independent at week 24 was defined as requiring RBC transfusions or hemoglobin levels < 8 g/dL during weeks 13 to 24. Transfusion dependence at week 24 was defined as requiring ≥ 4 units of RBCs or hemoglobin levels < 8 g/dL during weeks 17 to 24; not transfusion dependent at week 24 was defined as requiring < 4 units of RBCs and hemoglobin levels ≥ 8 g/dL during weeks 17 to 24. Originally presented at the American Society of Hematology 58th Annual Meeting [13]. IPSS, International Prognostic Scoring System; OS, overall survival; RBC, red blood cell. *Stratified by study, IPSS risk, and baseline anemia status

Overall survival was not significantly different between ruxolitinib-treated patients who were transfusion independent and not independent at week 24 (*P* = 0.1322; Fig. 2a, e [13]), whereas there was a statistically significant difference in the control-treated subgroups (*P* = 0.0004; Fig. 2b, f). Among patients who were not transfusion independent at week 24, median OS favored ruxolitinib versus control in those with baseline anemia (200 vs 137 weeks) and those without baseline anemia (271 vs 166 weeks; overall *P* = 0.002).

Overall survival in the ruxolitinib group was similar between patients who were transfusion dependent and not dependent at week 24 (*P* = 0.4547; Fig. 2 c, g), whereas there was a statistically significant difference in the control subgroups (*P* = 0.0323; Fig. 2 d, h). Among patients who were transfusion dependent at week 24, ruxolitinib versus control treatment prolonged OS in those with baseline anemia (191 vs 127 weeks) and those without baseline anemia (not reached vs 90 weeks; overall *P* = 0.0014).

## Discussion

This exploratory pooled analysis of the COMFORT trials demonstrated that long-term treatment with ruxolitinib prolonged survival compared with best available treatment or placebo in patients with int-2 or high-risk MF. Importantly, ruxolitinib treatment was associated with statistically significant improvements in OS irrespective of baseline anemia status or transfusion requirements at week 24. These findings agree with previous reports from the COMFORT trials [8, 9, 11].

Anemia and the resulting dependence on RBC transfusions have been associated with reduced OS in patients with MF [4, 5, 11, 14, 15]. Ruxolitinib treatment may cause an initial reduction in hemoglobin levels in some patients with MF; however, the levels typically stabilize within 24 to 36 weeks [9, 12]. Furthermore, a previous report demonstrated that ruxolitinib was associated with prolonged survival regardless of baseline anemia status [11]. The current analysis expanded on these findings by demonstrating that patients’ week 24 transfusion status did not significantly affect OS in the ruxolitinib group but was associated with reduced OS in the control group.

Although the survival benefit associated with ruxolitinib treatment in patients with int-2 or high-risk MF is well established, further improvements in patient outcomes may be achieved by limiting the cytopenias experienced by some patients during treatment initiation. A recent phase 2 study of patients with int-2 or high-risk MF assessed combination treatment with ruxolitinib and danazol to obviate ruxolitinib-related anemia and thrombocytopenia [16]. Hematologic stabilization was achieved in most patients; however, the trial was halted due to modest efficacy per International Working Group-Myeloproliferative Neoplasms Research and Treatment criteria, and results from only 14 patients were reported. Further research is necessary to identify strategies that may be able to limit the initial cytopenias associated with ruxolitinib treatment in some patients.

## Conclusions

Taken together, these findings indicate that anemia and transfusion status at week 24 do not affect the survival advantage of patients with int-2 or high-risk MF treated with ruxolitinib. Moreover, they underscore the importance of monitoring for cytopenias, which are generally manageable with ruxolitinib dose modifications [8, 10] and adjuvant therapy. Given these data, there is a rationale for exploring ruxolitinib earlier in the disease course to assess the effect on the natural history of MF.

## Abbreviations

HR: Hazard ratio
int-2: Intermediate-2
IPSS: International Prognostic Scoring System
ITT: Intent to treat
JAK: Janus kinase
MF: Myelofibrosis
OS: Overall survival
PET-MF: Post–essential thrombocythemia myelofibrosis
PMF: Primary myelofibrosis
PPV-MF: Post–polycythemia vera myelofibrosis
RBC: Red blood cell
RPSFT: Rank-preserving structural failure time
